# Socio‐economic differences in patient participation behaviours in doctor–patient interactions—A systematic mapping review of the literature

**DOI:** 10.1111/hex.12956

**Published:** 2019-08-09

**Authors:** Sarah Allen, Simon N. Rogers, Rebecca V. Harris

**Affiliations:** ^1^ Department of Health Services Research, Institute of Population Health Sciences University of Liverpool Liverpool UK; ^2^ Evidence‐Based Practice Research Centre (EPRC), Faculty of Health and Social Care Edge Hill University Ormskirk UK; ^3^ Consultant Regional Maxillofacial Unit University Hospital Aintree Liverpool UK

**Keywords:** communication, Doctor–patient relationship, inequalities, patient participation, socio‐economic status

## Abstract

**Background:**

The degree to which patients participate in their care can have a positive impact on health outcomes. This review aimed to map the current literature on patient participation behaviours in interactions with physicians and the extent to which differences in these behaviours can be explained by socio‐economic status (SES).

**Search strategy:**

Four electronic databases were searched from 1980 onwards using key words related to socio‐economic status and patient participation behaviours.

**Study selection:**

Titles, abstracts and full texts were screened by two reviewers, with the second reviewer screening 20% of all entries.

**Data extraction:**

Data on year of publication, country, patient population, setting, patient participation behaviour studied, and SES measure used were extracted.

**Main results:**

Forty‐nine studies were included in the review. Most studies were conducted in the United States, and the most commonly studied patient participation behaviour was involvement in decision making. Most studies measured SES using education as an indicator, with very few studies using occupation as a measure. Many studies did not report on participants’ medical condition or study setting. Patient participation in their health‐care appointment increased with increasing SES in 24 studies, although in 27 studies no significant association was found.

**Discussion and conclusions:**

Current literature was found to be mainly US‐centric. Many studies did not specify participants’ medical condition or in what setting the study was undertaken. More studies are needed on less commonly studied patient participation behaviours. It would be helpful for further studies to also include a wider range of SES indicators.

## 1 INTRODUCTION

Patient‐centred care has been associated with beneficial outcomes such as a greater adherence to treatment, satisfaction and improved quality of life.^1^, ^2^, ^3^, ^4^ The Institute of Medicine defines patient‐centred care as providing care that is respectful of and responsive to individual patient preferences, needs and values, ensuring that patient values guide all clinical decisions.^5^ Thus, the extent to which patients participate in discussions during their hospital or clinic visits is seen as an important barometer of patient‐centred care. Although there is no universally applied definition on what type of behaviours constitutes patient participation in clinical visits,^6^ most studies focusing on patient participation behaviours involve a range of behaviours such as question asking, raising concerns, and expressing opinions, preferences and emotions.^7^

Often ‘patient participation behaviours’ are described as a general group of behaviours that characterize doctor–patient communication, rather than describing in detail the different ways patient participation can be measured or other component parts of doctor–patient communication behaviour which are classified in a different way. For example, an important previous systematic review by Verlinde et al^8^ focused more globally on doctor–patient communication behaviours, with the electronic search terms based on ‘doctor–patient communication’ and ‘physician–patient relations’. The review reported evidence showing that a social gradient in doctor–patient communication exists and classified this according to the following classification: verbal behaviour including instrumental and affective behaviour, non‐verbal behaviour and patient‐centred behaviour. Although the review found that patients with low socio‐economic status (SES) tended to participate less actively in their care, the study and its search strategy were insufficiently sensitive to allow identification as to whether certain patient participation behaviours were more researched or more important than others, since the focus of the study was doctor–patient communication in general.

The Verlinde et al^8^ review also limited identification of literature exploring the social gradient in doctor–patient communication and social gradient, to studies reporting the ‘social class related concepts of’ educational level, income or occupation. Confusingly, three of the studies included in this review measured SES using ‘social class’, although the authors did not specify exactly how this was defined. However, there are several other indicators of SES which may also be associated with patient participation behaviours such as the patients’ health insurance status or receipt of benefits, and also area‐level measures of deprivation related to the patients’ home address (Indices of Multiple Deprivation), which may not have been captured previously, and may still be relevant.^9^ Bearing in mind the potential importance of this area and its likely relationship to beneficial health outcomes, we undertook a systematic mapping review to identify what research had been done which specifically examined how patient participation behaviours in doctor–patient interactions are related to differences in a wide range of possible measures of socio‐economic status.

We chose to conduct a systematic mapping review, as such reviews are useful for detecting patterns in a large body of literature in order to identify areas for future research. As such, details of the included studies are summarized without quality assessment or presenting statistical analyses.^10^, ^11^

## 2 PURPOSE

Our research question was as follows: How and why does tendency to and desire for patient participation behaviours in health‐care consultations with physicians vary according to SES and what measures of SES have been explored? For the purpose of this review, we defined patient participation behaviours as consisting of question asking, raising concerns, involvement in decision making, rapport building, and expression of opinions, preferences and emotions.

## 3 DATA SOURCES

An electronic search was undertaken of the following databases: Medline, CINAHL, PsychINFO and Web of Science. Literature was searched from 1980 to 2018; since prior to 1980, there was much less electronic indexing. A pilot search was conducted to identify potentially eligible papers, assess the amount of relevant literature in the field and identify suitable search terms. At this stage, we found that including screening appointments and emergency admissions made the scope of the review far too broad and unmanageable; therefore, we decided to introduce limits in the electronic search terms regarding ongoing doctor–patient relationships. The electronic search contained free text and subject headings including patient‐centred care, question asking, raising concerns, involvement in decision making, building rapport, expression of preferences, emotions or opinions, educational status, income, occupational status, employment, social class and socio‐economic factors. This was modified as necessary for each database and can be found in Appendix S1.

Inclusion criteria for the review were as follows:

- Studies involving patient perspectives on actual and desired question asking, raising concerns, involvement in decision making, rapport building, or expression of opinions, preferences and emotions.
- SES gradient measured in the form of education, income, occupation or ‘other measures’ which included patients’ health insurance status, income indicators of state benefits and area‐based measures relating to the patients’ home address.
- Published in 1980 onwards.
- Studies involving adult patients.
- Only studies which focused on doctor–patient interactions.
- Written in English language only.

Studies were excluded if:

- They included only health‐care professional perspectives on patient participation.
- Patients under 18 or parents of patients only were recruited.
- Adult patient perspectives of childhood experiences were collected.
- The study was conducted in a country on the OECDs Development Assistance Committee list of Official Development Assistance recipients.^12^ This was in order to limit literature to higher income countries where the health‐care systems were likely to be similar.
- The appointment involved emergency attendances or screening.
- The interactions were with health‐care professionals who were not medical doctors.
- They were opinion articles.
- They were systematic reviews.

## 4 STUDY SELECTION

One reviewer (SA) screened all titles and abstracts identified through electronic searches, and 20% of the entries were double screened by a second reviewer (DH). All full‐text articles were then screened by one reviewer (SA), and 20% of the full texts were double screened by a second reviewer (DH). If the two reviewers disagreed on any papers, this was resolved by discussion with two other independent reviewers (RH and SR).

## 5 DATA EXTRACTION

Data extraction was independently conducted by both reviewers and the following information was obtained: year published, country the study was conducted in, study method and design, population recruited, study setting, sample size, how SES is measured, what patient participation behaviours are reported and key results.

## 6 RESULTS

**Figure 1:**
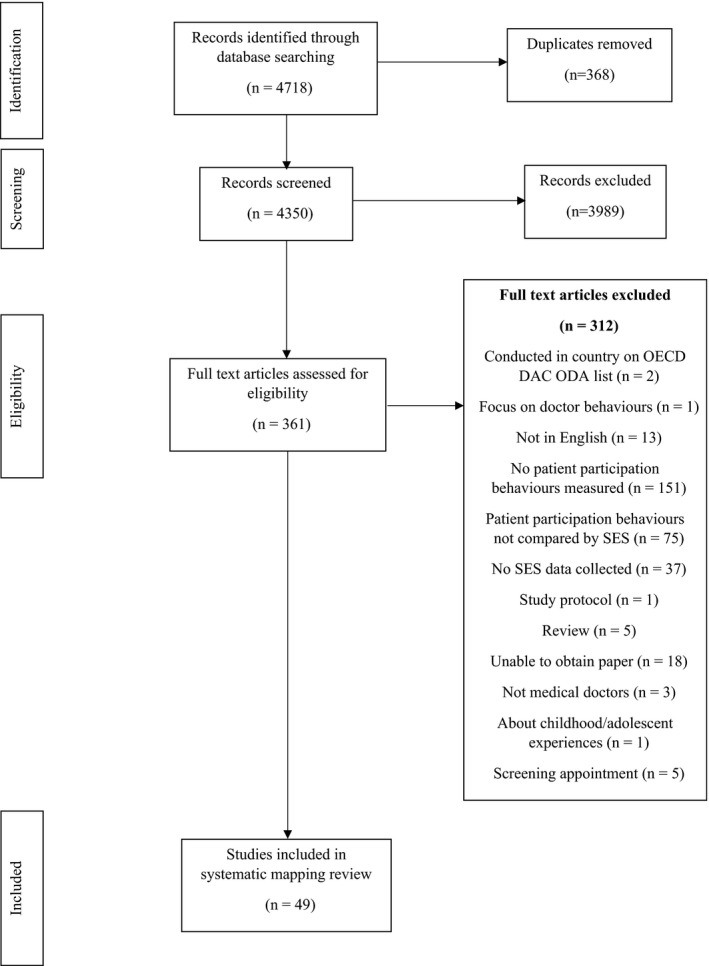
PRISMA diagram

The title and abstracts of 4718 articles were imported into Endnote, and 368 duplicates were removed. This left 4350 entries, of which 3989 articles were excluded leaving 361 entries. After screening all 361 full‐text articles, the two reviewers disagreed on 11 papers. Following discussion, seven papers were excluded. After screening, 49 studies were included in the review. The PRISMA diagram can be found in Figure 1.

**Figure 2:**
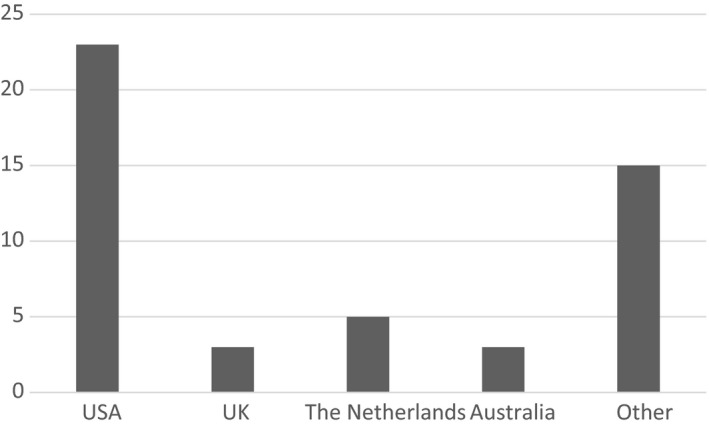
Countries the included studies were conducted in

Details of the characteristics of the 49 included studies can be found in Table 1. Overall, 39 (79.6%) of the included studies were published in the last 10 years, with only 10 being published before 2008. Most of the studies were conducted in the United States (46.9%), with the Netherlands being the second most common (10.2%). There were only three studies conducted in Australia, and only three conducted in the UK. ‘Other’ countries included Spain, Estonia, Germany, Norway and Finland (Figure 2).

**Table 1:** Characteristics of included studies and reported direction of association between socio‐economic status and patient participation behaviours

Author	Country		Study population	Methods	Number of participants	Socio‐economic status (SES) measure	Patient participation behaviours measured	Direction of association
1. Aasen et al (2012)^34^	Norway		End‐stage renal disease patients	Qualitative interviews	11	Education	Involvement in decision making, question asking, and expression of opinions	No statistical analyses performed
2. Ackermans et al (2018)^35^	The Netherlands		Patients with osteoarthritis of the hip or knee	Questionnaire	142	Education and employment	Involvement in decision making, and expression of opinions, preferences and emotions	No associations
3. Adams et al (2001)^26^	Australia		Asthma patients	Questionnaire	128	Income, education, employment, receipt of benefits, and housing situation	Involvement in decision making	Positive association with education only
4. AlHaqwi et al (2015)^36^	Saudi Arabia		Adult family practice patients	Questionnaire	236	Education	Involvement in decision making	Positive association
5. Aro et al (2012)^37^	Estonia		Adult ICU patients	Questionnaire	166	Education	Involvement in decision making	Negative association
6. Arora et al (2000)^38^	USA		Hypertension, diabetes, congestive heart failure, myocardial infarction and clinical depression patients	Questionnaire	2197	Education, income and employment	Involvement in decision making	Positive association with education only
7. Attanasio et al (2015)^39^	USA		Women aged 18‐45 who gave birth in US hospitals	Questionnaire	2400	Education and insurance	Question asking	Positive association for education Negative association for insurance type
8. Beauchamp et al (2015)^40^	Australia		Patients attending chronic disease services	Questionnaire	813	Insurance and education	Involvement in decision making	No associations
9. Bell et al (2001)^15^	USA		Patients reporting a new or worsening problem, or worries about serious illness	Questionnaire	909	Education, employment, income and insurance	Raising concerns	No association for education and income only, other SES variables not analysed
10. Bozec et al (2016)^23^	France		Head and neck squamous cell carcinoma patients	Questionnaire	200	Education and occupation	Expression of preferences	No associations
11. Chung et al (2012)^41^	USA		Patients admitted to a general internal medicine service	Questionnaire	8308	Education	Involvement in decision making and expression of preferences	Positive association for involvement in decision making only
12. Cohen et al (2013)^22^	USA		Patients admitted to hospital for hematopoietic stem cell transplantation	Longitudinal qualitative interviews	60	Education and occupation	Involvement in decision making	No statistical analyses performed
13. Dang et al (2017)^21^	USA		New patients attending a HIV clinic	Longitudinal qualitative interviews	21	Occupation	Question asking and involvement in decision making	No statistical analyses performed
14. De las Cuevas et al (2014)^42^	Spain		Outpatient psychiatric patients	Questionnaire	846	Education	Involvement in decision making	No associations
15. Deen et al (2011)^13^	USA		Community health centre patients	Intervention‐ pilot study	252	Education	Involvement in decision making	No associations
16. Durand et al (2016)^43^	UK		Chronic kidney disease patients	Questionnaire	492	Education	Involvement in decision making	No associations
17. Ellington et al (2006)^44^	USA		General population (some had cancer)	Focus groups	55	Education and employment	Involvement in decision making and expression of preferences	No statistical analyses performed
18. Friis et al (2016) 16	Denmark		Patients with diabetes, cardiovascular disease, COPD, musculoskeletal disorders, cancer, or mental disorders	Questionnaire	29,473	Education	Question asking, raising concerns, and expression of opinions, preferences and emotions	Positive associations
19. Garfield et al (2007)^27^	UK		Patients with type 2 diabetes or rheumatoid arthritis	Questionnaire	516	Social class (composite measure)	Involvement in decision making	Positive associations
20. Gleason et al (2016)^28^	USA		Older adults with hypertension, arthritis, cholesterol, diabetes, cancer, heart disease or depression	Questionnaire	277	Education, financial strain, and finances at the end of the month	Involvement in decision making	No significant associations
21. Henselmans et al (2015)^45^	The Netherlands		Patients diagnosed with a somatic chronic disease	Questionnaire	1314	Education	Involvement in decision making, question asking, and expression of opinions, preferences and emotions	No significant associations
22. Jacobs‐Lawson et al (2009)^46^	USA		Lung cancer patients	Questionnaire	100	Income and education	Involvement in decision making and expression of preferences	No significant associations for education only, income not entered into analysis
23. Janz et al (2004)^17^	USA		Breast cancer patients	Questionnaire	101	Education, employment and income	Involvement in decision making, question asking, raising concerns, and expression of opinions, preferences and emotions	Positive association between education and involvement in decision making only No significant associations for income and employment, and other participation behaviours not entered into analysis
24. Jonsdottir et al (2016) 47	Iceland		Patients who reported and consulted for chronic pain	Questionnaire	754	Education and income	Involvement in decision making	No significant associations
25. Lu et al (2011)^14^	USA		Underserved women newly diagnosed with breast cancer	Intervention‐ pilot study	231	Education	Involvement in decision making, question asking, and raising concerns	Positive association for question asking only No significant associations for other variables
26. Lubetkin et al (2010)^48^	USA		Patients attending urban health centres	Questionnaire	454	Education	Involvement in decision making	Positive association
27. Magnezi et al (2015)^19^	Israel		General population	Questionnaire	508	Education and income	Involvement in decision making, rapport building, and expression of preferences	Negative associations for rapport building and expression of preferences only Involvement in decision making not entered into analysis
28. Maly et al (2008)^18^	USA		Breast cancer patients	Questionnaire	257	Education and income	Question asking, raising concerns, involvement in decision making, and expression of opinions, preferences and emotions	Positive associations
29. Manderbacka (2005)^20^	Finland		Coronary heart disease patients	Qualitative interviews	30	Occupation and employment	Involvement in decision making	No statistical analyses performed
30. Mercer et al (2016)^24^	UK (Scotland)		Patients attending a GP practice	Questionnaire	659	Scottish Indices of Multiple Deprivation	Involvement in decision making	Positive association
31. Moise et al (2017)^49^	USA		Patients with uncontrolled hypertension	Questionnaire	195	Education and insurance	Involvement in decision making	Positive association for education only
32. Moret et al (2017)^25^	France		Gynaecology, orthopaedic, internal medicine, and emergency medicine hospital inpatients	Questionnaire	255	Deprivation (EPICES score and perceived social status), education, and employment	Involvement in decision making	Positive association for deprivation only Other variables not entered into analysis
33. Morishige et al (2017)^50^	Japan		Inflammatory bowel disease patients	Questionnaire	1035	Income, education and employment	Involvement in decision making	No associations
34. Morrison et al (2003) 51	Australia		General population	Questionnaire	1297	Education and income	Involvement in decision making, and expression of preferences	Negative associations
35. Murray et al (2007)^52^	USA		General population	Questionnaire	3177	Education, income and insurance	Involvement in decision making	Positive associations for education and income only
36. Nijman et al (2014)^53^	The Netherlands		General population	Questionnaire	1432	Education and income	Involvement in decision making	Positive associations
37. Olson et al (2010)^54^	USA		Hospital inpatients	Questionnaire	89	Education and insurance	Involvement in decision making	No associations
38. Overgaard et al (2012)^55^	Denmark		Low risk women receiving midwifery unit or obstetric unit care	Questionnaire	375	Education and employment	Involvement in decision making	No associations
39. Phipps et al (2008) 56	USA		African American cancer patients who received chemotherapy	Questionnaire	26	Income and education	Involvement in decision making	No associations
40. Rademakers et al (2012)^57^	The Netherlands		Patients with rheumatoid arthritis, spinal disc herniation, or malignant or benign breast abnormalities	Questionnaire	1019	Education	Involvement in decision making and question asking	Positive associations
41. Skolasky et al (2011)^58^	USA		Community dwelling multimorbid adults	Questionnaire	855	Education and income	Involvement in decision making	Positive association for education only
42. Smith et al (2016)^59^	USA		General population	Questionnaire	3400	Income and education	Involvement in decision making	Positive associations
43. Spies et al (2006)^60^	Germany		Patients attending a chronic pain clinic	Questionnaire	341	Income, employment and education	Involvement in decision making and question asking	Positive associations for education only
44. Stepleman et al (2010)^61^	USA		Multiple sclerosis patients	Questionnaire	199	Education and employment	Involvement in decision making	Positive associations
45. Tariman et al (2014)^62^	USA		Symptomatic myeloma patients	Questionnaire	20	Employment, education, and income	Involvement in decision making	No associations
46. Tsimtsiou et al (2014)^63^	Greece		Hospitalized patients	Questionnaire	454	Education and income	Involvement in decision making and question asking	Positive associations for education only Income not entered into analysis
47. van den Brink‐Muinen et al (2011)^64^	The Netherlands		Patients diagnosed with a somatic chronic disease	Questionnaire	2423	Education	Involvement in decision making	No association
48. Yek et al (2017)^65^	Singapore		Patients attending a pre‐operative evaluation clinic for elective surgical procedures	Questionnaire	364	Education, employment, insurance and income	Involvement in decision making and question asking	Positive associations for education, employment, and insurance only Income not entered into analysis
49. Yeo (2016) 66	USA		General population	Questionnaire	2297	Education, employment, income and insurance	Involvement in decision making and question asking	Negative associations for education and income only Positive associations for insurance Employment not entered into analysis

**Figure 3:**
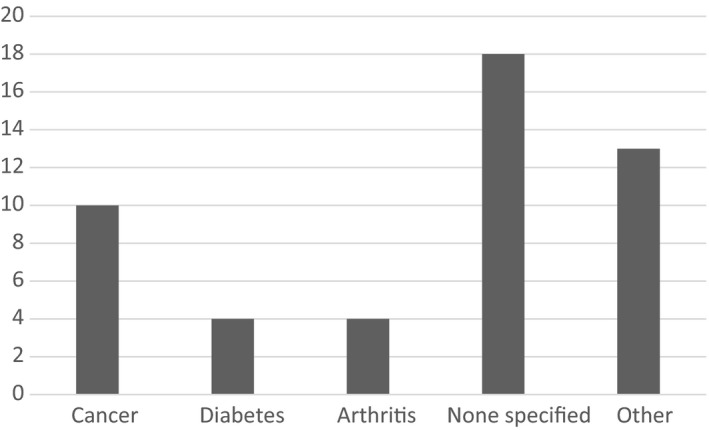
Diagnoses of recruited participants

The majority of studies used questionnaires to collect data (75.5%), with only five studies using qualitative techniques such as interviews or focus groups, and only two studies 13, 14 were interventions. Both interventions were pilot studies with no control group. The most commonly studied condition was cancer (20.4%), with four studies recruiting arthritis patients, and four studies with diabetes patients. Most studies did not specify what condition (if any) their participants had (36.7%). ‘Other’ conditions included asthma, chronic pain, HIV, multiple sclerosis and inflammatory bowel disease (Figure 3). None of the three UK studies recruited cancer patients.

The most common setting for studies involved secondary or tertiary care (44.9%), with primary care being the setting in only 11 studies. Unfortunately, 16 studies did not specify which setting their research referred to when collecting data from participants. The most commonly studied patient participation behaviour was involvement in decision making (46 studies), whereas five studies examined raising concerns,^14^, ^15^, ^16^, ^17^, ^18^ and only one study looked at rapport building.^19^ Question asking and expression of opinions, preferences or emotions was more commonly studied, featuring in 13 and 12 studies, respectively. The rapport building study recruited participants from the general population and so there was a lack of studies which focused on rapport building which involved participants in a health setting. Three of the raising concerns studies were with breast cancer patients (Table 2).

**Table 2:** Summary of SES variables and patient participation behaviours used in included studies

	Patient participation behaviours				
	Involvement in decision making	Question asking	Raising concerns	Rapport building	Expression of opinions, preferences or emotions
SES measure					
Education	1, 2, 3, 4, 5, 6, 8, 11, 12, 14, 15, 16, 17, 20, 21, 22, 23, 24, 25, 26, 27, 28, 31, 32, 33, 34, 35, 36, 37, 38, 39, 40, 41, 42, 43, 44, 45, 46, 47, 48, 49	1, 7, 18, 21, 23, 25, 28, 40, 43, 46, 48, 49	9, 18, 23, 25, 28	27	1, 2, 10, 11, 17, 18, 21, 22, 23, 27, 28, 34
Employment	2, 3, 6, 17, 23, 29, 32, 33, 38, 43, 44, 45, 48, 49	23, 43, 48, 49	9, 23		2, 17, 23
Income	3, 6, 22, 23, 24, 27, 28, 33, 34, 35, 36, 39, 41, 42, 43, 45, 46, 48, 49	23, 28, 43, 46, 48, 49	9, 23, 28	27	22, 23, 27, 28, 34
Occupation	12, 13, 29	13			10
Insurance	8, 31, 35, 37, 48, 49	7, 48, 49	9		
Deprivation	30, 32				
Receipt of benefits	3				
Housing situation	3				
Social class	19				
Financial strain	20				
Finances at end of month	20				

Note

Each study in Table 1 was assigned a number, which corresponds with the numbers in this tab.

The most commonly used measure to explore relationships between patient participation behaviours and SES was educational level (45 studies). Only four studies used occupation as an indicator of SES,^20^, ^21^, ^22^, ^23^ and only two studies used a composite measure of deprivation.^24^, ^25^ One study measured housing situation and receipt of benefits,^26^ one used a composite measure of social class,^27^ and one looked at financial strain and finances at the end of the month.^28^ It is also important to note that many studies examined more than one type of patient participation behaviour or used more than one measure of SES. Most studies (33) used more than one measure of SES, whereas only 18 studies examined more than one type of patient participation behaviour (Table 2).

Table 1 shows the direction of associations reported in the included studies. Of the 49 included studies, 5 did not perform statistical analyses as they had employed qualitative methodologies. Positive associations between SES and patient participation behaviours (PPBs) were reported by 24 studies, while 5 studies reported negative associations. Twenty‐seven studies reported no association between at least some of their variables. Eleven studies reported associations of differing directions for different measures of SES or different PPBs within their study. Since the studies were so mixed in terms of design, participants’ condition, outcomes and setting, it was not appropriate to undertake any meta‐analyses.

Of the 23 studies conducted in the United States, 13 reported a positive association between SES and PPB, 2 reported a negative association, and 14 reported no association between at least some of their variables. Three studies did not perform statistical analyses, and 9 studies reported associations of differing directions for different measures of SES and PPB.

Of the 10 studies conducted with cancer patients, 4 reported a positive association between SES and PPB, 7 reported no association between at least some of their variables, and 2 reported associations of differing directions for different measures of SES and PPB. One study did not perform statistical analyses.

## 7 DISCUSSION

Although the goal of systematic review searches is to identify all relevant studies on a topic, it is necessary to balance comprehensively covering a topic (or sensitivity of a search) with how manageable it is within resources available.^11^ On the other hand, a wider search may reduce precision (identifying non‐relevant articles), which while more comprehensive, may be more difficult to summarize because types of studies may vary quite widely. Systematic mapping reviews help by a method to overview a larger area so that gaps to inform future research can be identified.^10^, ^11^

Our study shows that while an earlier systematic review exploring literature on the social gradient in doctor–patient communication had a relatively broad search strategy, this included only 20 papers,^8^ whereas our study focusing purely on patient participation behaviours and SES differences identified 49 studies. Although this may indicate an expanding area of research, this may also be because our study used a wider set of SES indicators than had been used previously. Our research is particularly informative because it focused in detail on the patient‐side of the clinical interactions, whereas other reviews have had a main focus on behaviours in the consultation.^8^, ^29^

We found that the most commonly used measure of SES in studies of this type was educational level, while measures of participants’ occupation have been much less frequently used. Income and employment status were not as commonly measured as educational level, although they were still used in some studies. Occupation is a key indicator of SES and likely to have an important influence on the doctor–patient relationship,^30^ and so it is surprising to find so few previous studies using this measure.

We found that the most frequently studied patient participation behaviour was involvement in decision making, whereas raising concerns and building rapport were comparatively relatively neglected. In contrast, Verlinde et al 8 found fewer studies on joint decision making and a larger number of studies involving other types of patient participation behaviours. Perhaps patient‐orientated communication studies have had more focus on decision‐making aspects of communication, whereas doctor‐orientated communication studies focus on other aspects of the relationship—or our more specific electronic search terms which included ‘decision making’, meant that we could better reflect the amount of research which has been undertaken in this field.

Although previous studies have found that rapport building in the doctor–patient relationship can have a number of positive outcomes, including treatment satisfaction, understanding health information, coping and adherence to treatment,^1^, ^3^, ^31^ only one study was identified which looked at how this behaviour was related to SES difference, and so further research in this area is particularly needed.

Most studies used more than one measure of SES which in some cases allowed a comparison of the effects of each different measure, although in some of these, not all the SES variables were entered into the analysis but were simply used to describe the sample. The objective of our study was to map the literature in this area rather than to produce a synthesis across several types of studies; however, we extracted data from included studies on whether a statistically significant association between SES and PPB had been reported. This indicated that although PPB was found to be related to SES in about half of the studies, in about half, they were not. Summarizing results are made more difficult by the heterogeneity which exists between studies in this area, and the range of different measures of SES and indicators of PPB which had been used. For example, although several studies showed an association with education and patient participation behaviours, as many as 17 studies found no statistically significant association between the two variables; and so the relationship is likely to be complex. On the other hand, few studies seem to have found a significant association between patient participation behaviours and employment or income. Larger and more sophisticated studies are needed, using a range of SES indicators and a more in‐depth description of patient participation behaviours, and the setting involved.

While the most common condition studied was cancer and the most common setting was secondary or tertiary care, 36.7% of studies did not specify what condition (if any) their participants were diagnosed with or what health‐care setting their questions regarding patient participation referred to. This is potentially important information which is missing from these studies, as setting and condition which the patient is consulting for can influence a patient's preferred and experienced level of participation in a consultation.^7^, ^32^, ^33^

Most studies included in the review were conducted in the United States, making the current research in this area very US‐centric. This may limit the generalizability of the results of these studies, as other countries have differently structured health‐care systems which might influence patient participation behaviours. There is a need for more studies on patient participation behaviours outside of the United States.

## 8 CONCLUSION

In conclusion, our findings suggest that most patient participation research relies on education as an indicator of SES and mainly explores involvement in decision making as the patient participation behaviour of interest. Most previous studies have been undertaken in the United States, but many lack important information on the setting or the patients’ condition. More studies on specific patient participation behaviours such as rapport building and raising concerns are needed, and other studies undertaken outside the United States. Use of a wider range of SES measures such as occupation, housing situation, receipt of benefits and household finances would be useful additional data.

## CONFLICTS OF INTEREST

None.

## Data Availability

Data sharing is not applicable to this article as no new data were created or analysed in this study.
