# Supplementary material for: Socio‐economic differences in patient participation behaviours in doctor–patient interactions—A systematic mapping review of the literature
Source: Health Expect. 2019 Aug 9;22(5):1173–84. doi: 10.1111/hex.12956 (PMC6803421; doi:10.1111/hex.12956)
Supplement: Supplementary file 1 [file HEX-22-1173-s001.docx]

**Appendices**

Appendix 1. Medline search strategy (4104 results)

| [# ▲](http://ovidsp.tx.ovid.com.liverpool.idm.oclc.org/sp-3.28.0a/ovidweb.cgi?&S=NFOMFPOOHKDDLGNBNCFKEBLBLIHDAA00&Sort+Sets=descending) | **Searches** | **Results** |
| --- | --- | --- |
| 1 | Chronic Disease/ | 246072 |
| 2 | (chronic adj3 (diseas* or condition* or medical*)).tw. | 244359 |
| 3 | Patient-Centered Care/ | 15618 |
| 4 | ((patient* or health*) adj3 (empower* or percept* or perspective* or activat*)).tw. | 59435 |
| 5 | ((ongoing or continu*) adj3 care*).tw. | 20788 |
| 6 | or/1-5 | 537049 |
| 7 | exp *Educational Status/ | 7671 |
| 8 | exp *Socioeconomic Factors/ | 146986 |
| 9 | exp *Health Status Disparities/ | 7604 |
| 10 | exp *Income/ | 28082 |
| 11 | exp *Employment/ | 41673 |
| 12 | exp *Social Class/ | 12148 |
| 13 | socioeconomic*.mp. | 188474 |
| 14 | disadvantaged.mp. | 10832 |
| 15 | deprived.mp. | 25919 |
| 16 | "low income".mp. | 28098 |
| 17 | "educational status".mp. | 49215 |
| 18 | "occupational status".mp. | 2021 |
| 19 | ((poverty or income or educational* or occupation* or "low income" or social) adj2 (analysis or disadvantage* or specific or difference* or factor* or inequalit* or depriv* or inequit* or disparit*)).mp. | 37356 |
| 20 | ((occupation* or income* or education* or social) adj3 (grade* or level* or status)).mp. | 114411 |
| 21 | Vulnerable Populations/ | 8334 |
| 22 | (vulnerable* adj2 (patient* or populat*)).tw. | 10738 |
| 23 | or/7-22 | 461469 |
| 24 | (question* adj4 (ask* or query or queries or enquir*)).tw. | 22310 |
| 25 | (rais* adj4 (concern* or query or queries)).tw. | 21046 |
| 26 | (involv* adj4 decision*).tw. | 7691 |
| 27 | (build* adj4 rapport*).tw. | 400 |
| 28 | (express* adj4 (opinion* or prefer* or emotion*)).tw. | 22858 |
| 29 | Professional-Patient Relations/ or Decision Making/ or "surveys and questionnaires"/ or patient health questionnaire/ or Patient Preference/ | 489889 |
| 30 | ((ongoin* or continu*) adj3 relationship*).tw. | 2063 |
| 31 | or/24-30 | 553148 |
| 32 | 6 and 23 and 31 | 4448 |
| 33 | (exp Child/ or Adolescent/ or exp Infant/) not exp Adult/ | 1726772 |
| 34 | 32 not 33 | 4145 |
| 35 | Animals/ not Humans/ | 4394059 |
| 36 | 34 not 35 | 4144 |
| 37 | Developing Countries/ | 69382 |
| 38 | 36 not 37 | 4119 |
| 39 | limit 38 to yr="1980 -Current" | 4104 |
